# SiC free-standing membrane for X-ray intensity monitoring in synchrotron radiation beamlines

**DOI:** 10.1107/S1600577524010646

**Published:** 2025-01-01

**Authors:** Gabriele Trovato, Marzio De Napoli, Christian Gollwitzer, Simone Finizio, Michael Krumrey, Francesco La Via, Luca Lanzanò, Giuliana Milluzzo, Samuele Moscato, Matthias Müller, Francesco Romano, Dario Ferreira Sanchez, Massimo Camarda

**Affiliations:** a Università degli Studi di Catania Dipartimento di Fisica e Astronomia ‘Ettore Majorana’ Via Santa Sofia 64 95123Catania Italy; b STLab srl, Via Anapo 53, 95126Catania, Italy; c Istituto Nazionale di Fisica Nucleare – INFN Sezione di Catania Via S. Sofia 64 95123Catania Italy; d Istituto per la Microelettronica e Microsistemi CNR-IMM Sezione di Catania Strada VIII Zona Industriale 5 95121Catania Italy; e SenSiC GmbH, DeliveryLAB, 5234 Villigen, Switzerland; f Physikalisch-Technische Bundesanstalt (PTB) Abbestraße 2-12 10587Berlin Germany; g Swiss Light Source Paul Scherrer Institut (PSI) Villigen Switzerland; h Università degli Studi di Catania Dipartimento di Ingegneria Elettrica, Elettronica e Informatica (DIEEI) Viale Andrea Doria 6 I-95125Catania Italy; Bhabha Atomic Research Centre, India

**Keywords:** silicon carbide, diamond, free-standing membranes, diagnostics, X-ray intensity monitoring, semiconductor detectors, beam transmittance, charge collection efficiency, responsivity

## Abstract

For many synchrotron radiation experiments, it is critical to perform continuous, real-time monitoring of the X-ray flux for normalization and stabilization purposes. Traditional transmission-mode monitors included metal mesh foils and ionization chambers, which suffered from low signal stability and size constraints. Solid-state detectors are now considered superior alternatives for many applications, offering appealing features like compactness and signal stability. However, silicon-based detectors suffer from poor radiation resistance, and diamond detectors are limited in scalability and are expensive to produce. Silicon carbide (SiC) has recently emerged as an alternative to both materials, offering a high-quality mature semiconductor with high thermal conductivity and radiation hardness. This study focuses on a systematic exploration of the SiC ‘free-standing membrane’ devices developed by SenSiC GmbH. In particular, we performed in-depth sensor-response analysis with photon energies ranging from tender (1.75 keV) to hard (10 keV) X-rays at the Four-Crystal Monochromator beamline in the PTB laboratory at the synchrotron radiation facility BESSY II, studying uniformity of transmission and responsivity compared with the state-of-the-art beam monitors. Furthermore, we theoretically evaluated the expected signal in different regions of the sensors, also taking into account the effect of charge diffusion from the SiC substrate in the case of the not-carved region.

## Abstract 2

Characterization of an SiC free-standing membrane to be used as an X-ray beam intensity monitor.

## 1. Introduction

Many synchrotron radiation experiments require precise X-ray beam monitoring to measure the flux of incident photons (Keister *et al.*, 2018 ▸). Initially, metal mesh foils were used, but the signal produced was small and unstable (Krumrey *et al.*, 2007 ▸). Ionization chambers represent another option (Ahmed *et al.*, 2000 ▸). They guarantee high accuracy and low beam attenuation, but need high voltage. In addition, ionization chambers are usually several centimetres long, in a context where the space used for such devices is critical and has to be taken into account in the design of the beamline. Moreover, they require gas at a certain pressure and thus also windows when operated in vacuum beamlines.

Another alternative technology for real-time beam intensity monitors is given by solid-state detectors. Due to the technological maturity of the semiconductor industry, very thin devices could be manufactured which allow real-time measurements without significantly altering the X-ray beam. Silicon diodes have been investigated as intensity beam monitors (Krumrey *et al.*, 2007 ▸; Owen *et al.*, 2009 ▸), but their limited radiation resistance constrains their extended use under high radiation dose. Diamond, in particular scCVD (single-crystal grown by chemical vapour deposition), is the favoured material for this type of application (Desjardins *et al.*, 2013 ▸; Houghton *et al.*, 2023 ▸). In fact, along with a relatively high X-ray transmission efficiency, owing to the low *Z* number of carbon atoms, this material guarantees a high radiation resistance and a high thermal conductivity (Nida *et al.*, 2019 ▸). An attempt to obtain ultra-thin (<20 µm) devices to perturb the beam as little as possible for synchrotron applications was made by Desjardins *et al.* (2014 ▸) using the reactive ion etching (RIE) technique to reduce the thickness of the scCVD detector: initially 60 µm thick down to 3 µm. However, the resulting device showed a highly non-homogeneous surface (>50%). A later paper from the same group (Desjardins *et al.*, 2017 ▸) reported a Schottky junction diode with a 4 µm diamond layer, with better surface uniformity. However, the main issue of diamond sensors is related to the lack of scalability in the production process, which prevents the fabrication of devices with an area larger than 1 cm^2^. Moreover, this type of detector suffers from critical deep-level defects (Weiss *et al.*, 2022 ▸) and problems related to material doping control (Medina *et al.*, 2023 ▸). For these reasons, silicon carbide (SiC) has recently attracted increasing interest as a potential alternative to diamonds. This wide band-gap semiconductor (3.26 eV for 4H-SiC) exhibits strong radiation resistance and a high thermal conductivity, both essential for a beam intensity monitor (Bertuccio & Casiraghi, 2003 ▸; De Napoli, 2022 ▸; Kimoto & Cooper, 2014 ▸). In addition, large-area wafers (up to 6′′) can be produced to realize devices with characteristics similar to diamond detectors but with larger sensitive areas and lower costs (SenSiC, 2024 ▸). What has so far hindered use of SiC in real-time monitoring applications is the almost ten times higher X-ray absorption coefficient compared with diamond due to the presence of ‘high-*Z*’ silicon atoms. For this reason, to achieve the same transmittance of diamond, the SiC sensors need to be thinned up to a factor ten compared with diamond requiring, for example, a sensor-thicknesses of less than 10 µm for photon energies <30 keV.

In order to optimize such detector structures and increase transmittance, the SenSiC GmbH start-up has been developed and is now commercializing SiC ‘free-standing membrane’ devices, which are – as originally reported by Nida *et al.* (2019 ▸) – obtained through an innovative electrochemical, doping-selective, etching process of the substrate on the back side of the device. Such technology was already presented as an X-ray beam position monitor device by Houghton *et al.* (2023 ▸) and a single ion detector in ion-implantation process by Sangregorio *et al.* (2023 ▸).

This study reports the characterization of this SiC free-standing membrane used, for the first time, as an X-ray beam intensity monitor in a synchrotron radiation facility, performing an extensive signal analysis as a function of the photon energy and in different detector regions to highlight the role of the free-standing membrane and of the doping profiles on the sensors’ functionality.

## 2. Materials and methods

### 2.1. Experimental setup

**Figure 1:**
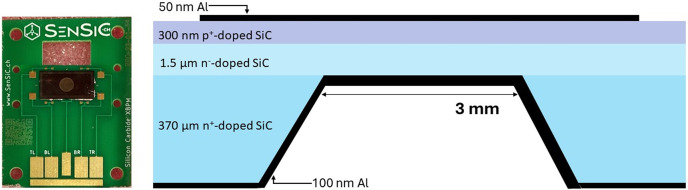
Image (left) and a schematic section (right) of the SiC detector: a ‘free-standing membrane’ based on a p–n junction (layer thicknesses are not on scale).

The device studied in this work, manufactured by the SenSiC GmbH start-up, is shown in Fig. 1 ▸. The device is an SiC p–n junction consisting of a 300 nm p^+^ layer (1 × 10^18^ cm^−3^), a 1.5 µm n^−^ epitaxial layer (1 × 10^14^ cm^−3^) and a 370 µm n^+^ (1 × 10^18^ cm^−3^) SiC substrate. The ‘free standing membrane’ is obtained in the central region of the 4 mm × 9 mm device through a process of doping-selective electrochemical etching (Nida *et al.*, 2019 ▸), which eroded the thick n^+^ layer in a circular window 3 mm in diameter, up to the n^−^ region. After this carving process, thin Al layers are deposited to form electrodes, and thus a rapid thermal annealing process is performed at 400°C for 2 min in argon. The back contact on the membrane is realized on contacting the aluminium to the n^−^ layer, forming a Schottky barrier.

**Figure 2:**
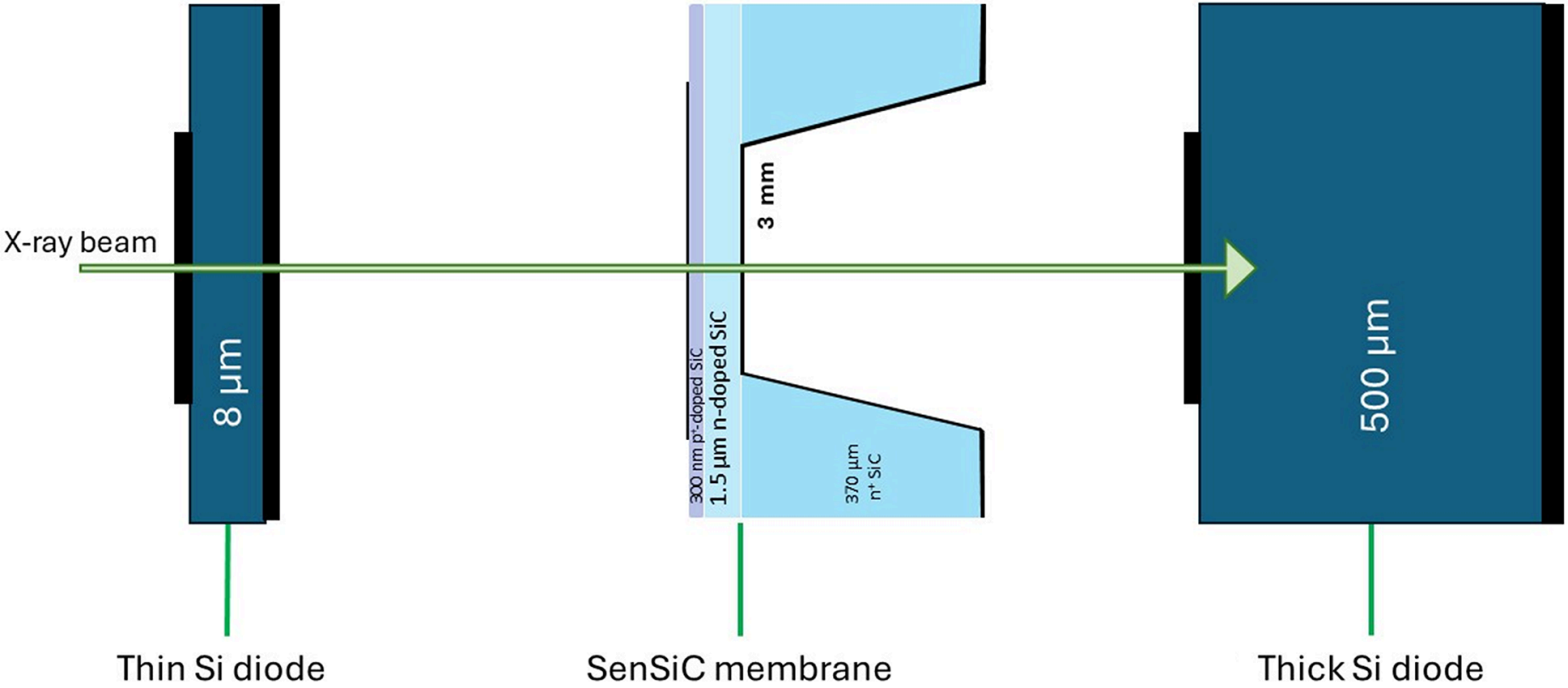
Schematic of the sequence of diodes into the beamline.

The study of the detector performance has been conducted at the BESSY II synchrotron radiation facility in Berlin, on the Physikalisch-Technische Bundesanstalt (PTB) Four-Crystal Monochromator beamline (Krumrey & Ulm, 2001 ▸). X-rays with energies from 1.75 keV to 10 keV (10^−4^ relative resolution) were used. The device has been placed inside a vacuum chamber on a motorized stage together with a thick (500 µm) Canberra silicon diode (PD50-500 CB), located downstream with respect to the beam direction (see Fig. 2 ▸). Upstream of these detectors, a thin (8 µm) custom-made Hamamatsu Si diode was placed as the monitor detector. They were used as a reference to extract certain properties of the SiC sensor, as discussed later. The two Si diodes were connected to two Keysight B2985A electrometers, while the SenSiC device was connected to a Keithley 617 electrometer. All the measurements were conducted without applying any bias to the SiC sensor. With such a setup, we were able to precisely measure the SiC transmission and responsivity as a function of the photon energy. In addition, an X-ray raster scan, with a step size of 100 µm and a beam spot of about 250 µm diameter, allowed us to perform 2D mapping of the collected current, both inside the area of the membrane and outside. From this measurement it was possible to extract 2D maps of transmission, charge collection efficiency (CCE) and responsivity.

### 2.2. Signal formation

A study of the current signal produced by the device during irradiation has been performed on both the free-standing membrane region and the not-carved region, where the n^+^ substrate is present (Fig. 2 ▸). The first step of our analytical formulation of the signal was to estimate the number of photons absorbed in each layer using the Beer–Lambert law (IUPAC, 2019 ▸),
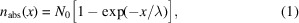
where *n*_abs_(*x*) is the number of X-ray photons absorbed at a depth *x* in the device, *N*_0_ the number of incident X-rays and λ the attenuation length, which is about 70 µm in SiC for X-rays of *E* = 8 keV (Henke *et al.*, 1993 ▸). The number of photons absorbed is then converted into the number of electron–hole pairs generated using the relation
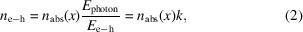
where *E*_e−h_ = 7.6 eV in 4H-SiC (De Napoli, 2022 ▸) is the electron–hole creation energy and *E*_photon_ is the energy of the photons in electronvolts.

Using this latter equation, it is possible to estimate the theoretical drift current generated in the membrane (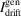). The average photon flux incident on the membrane (1.89 × 10^8^ s^−1^) reduces to the value 1.88 × 10^8^ s^−1^ due to absorption in the Al electrode and the p^+^ layer. This is therefore the photon flux that reaches the n^−^ layer, assumed to be fully depleted even though no bias is applied. Indeed, according to the relation 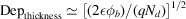, where *q* is the electron charge and ε is the SiC dielectric constant, for a doping concentration *N* = 1 × 10^14^ cm^−3^, a built-in potential of ϕ_b_ = 0.2 V is already enough to fully deplete a thickness of Dep_thickness_ = 1.5 µm. Thus, considering the number of photons absorbed in the 1.5 µm thickness of the n^−^ layer from equation (2), we obtain 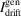 = 0.67 nA. This current would match the measured one if CCE = 100%.

When the beam hits the device region with the substrate (*i.e.* the not-carved region), it is possible that a portion of the minority charge carriers generated by radiation in the substrate will diffuse and reach the junction region where the electric field is present, and is then collected. This contribution has been estimated in the following manner. It is assumed that an exponential term of the type 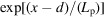 accounts for the probability of a minority carrier, generated at a depth *x*, to reach the edge of the depletion region. This is regardless of the mechanism by which the charge was produced, that is, due to ionization induced by a charged particle (Breese, 1993 ▸; De Napoli *et al.*, 2009 ▸) or through interaction with electromagnetic radiation, as in the present case. The decreasing exponential probability is governed by the diffusion length *L*_p_ (*i.e.* the average distance that a minority carrier diffuses before it recombines with charges of the opposite sign). The depth of the interface between the substrate and the epitaxial region *d* = 1.8 µm. Thus, it is further assumed that the depletion region has an abrupt boundary and that the depletion layer thickness is equal to the *n*-thickness, as previously discussed. Based on the above assumptions, the minority charge carriers that reach the electric field region can be estimated as
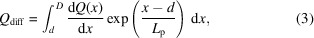
where *D* = 371.8 µm denotes the depth at which the substrate ends and d*Q*(*x*)/d*x* is the infinitesimal charge generated at a depth *x*. The infinitesimal charge can be expressed using equations (1) and (2),
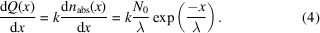
By inserting this expression into the integral of equation (3) and defining a new variable as 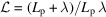, the integral takes the known form 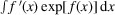. This can be solved analytically, giving the final result
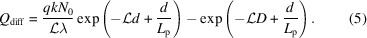
The current in the not-carved region (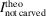) is thus given by the sum of the drift current (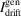, previously calculated) and the current due to those charges that, through diffusion, manage to reach the depleted zone [*I*_diff_, calculated using equation (5)], *i.e.*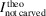 = 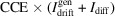. The current experimentally measured in the not-carved region is about twice that of the membrane (see the next section) and can be reproduced with a value of *I*_diff_ = 0.616 nA, considering the experimental CCE of 86%. This *I*_diff_ value is obtained from equation (5) using *L*_p_ = 1.4 µm. Note that the extra charge measured in the region with the substrate might not be entirely due to diffusion if *L*_p_ is smaller than the assumed value. A contribution could come also from the presence of a contact potential induced on the back of the membrane by the Al contact (which is not present in the thicker part of the device) and therefore to a different extension of the electric fields in the two regions.

## 3. Results and discussion

### 3.1. Detector characterization

**Figure 3:**
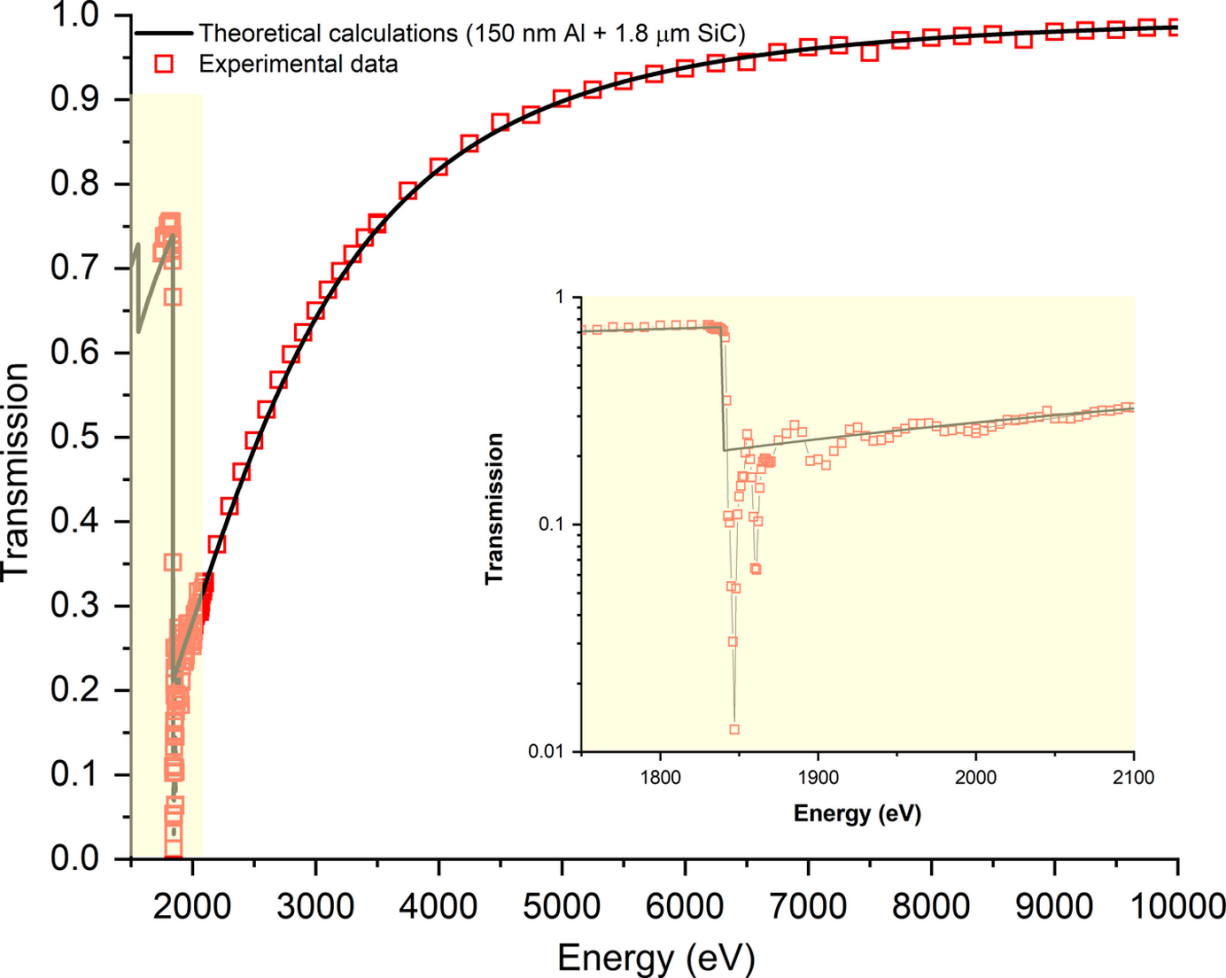
Transmission coefficient of the SiC membrane as a function of X-ray energy. Data are compared with theoretical calculations (Henke *et al.*, 1993 ▸).

The transmittance of the device is obtained by measuring the current produced by both the thin and the thick Si diodes, *I*_thin_ and *I*_thick_. The measurement is performed twice, with and without the SiC device between the Si detectors. With *r*_SiC_ and *r*_NOSiC_, the ratio of the currents *I*_thick_/*I*_thin_ in both scenarios, it is possible to extract the transmittance as
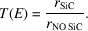
Fig. 3 ▸ shows the transmittance of the free-standing membrane as a function of X-ray energy. Near the Si *K*-edge, the typical absorption peak and the peculiar undulatory behaviour of EXAFS (extended X-ray absorption fine structure) are observed (Eisenberger & Kincaid, 1978 ▸). As expected, the transmittance increases with increasing X-ray energy, reaching values exceeding 90% for energies above 5 keV. The experimental results are compared with theoretical calculations obtained using absorption cross-sections of SiC and Al layers from Henke *et al.* (1993 ▸). The agreement is good, except near the *K*-edge region (insert of Fig. 3 ▸). This discrepancy could be attributed to the difference between a real layered material and the single atoms considered in the calculations (Hopman *et al.*, 2012 ▸).

**Figure 4:**
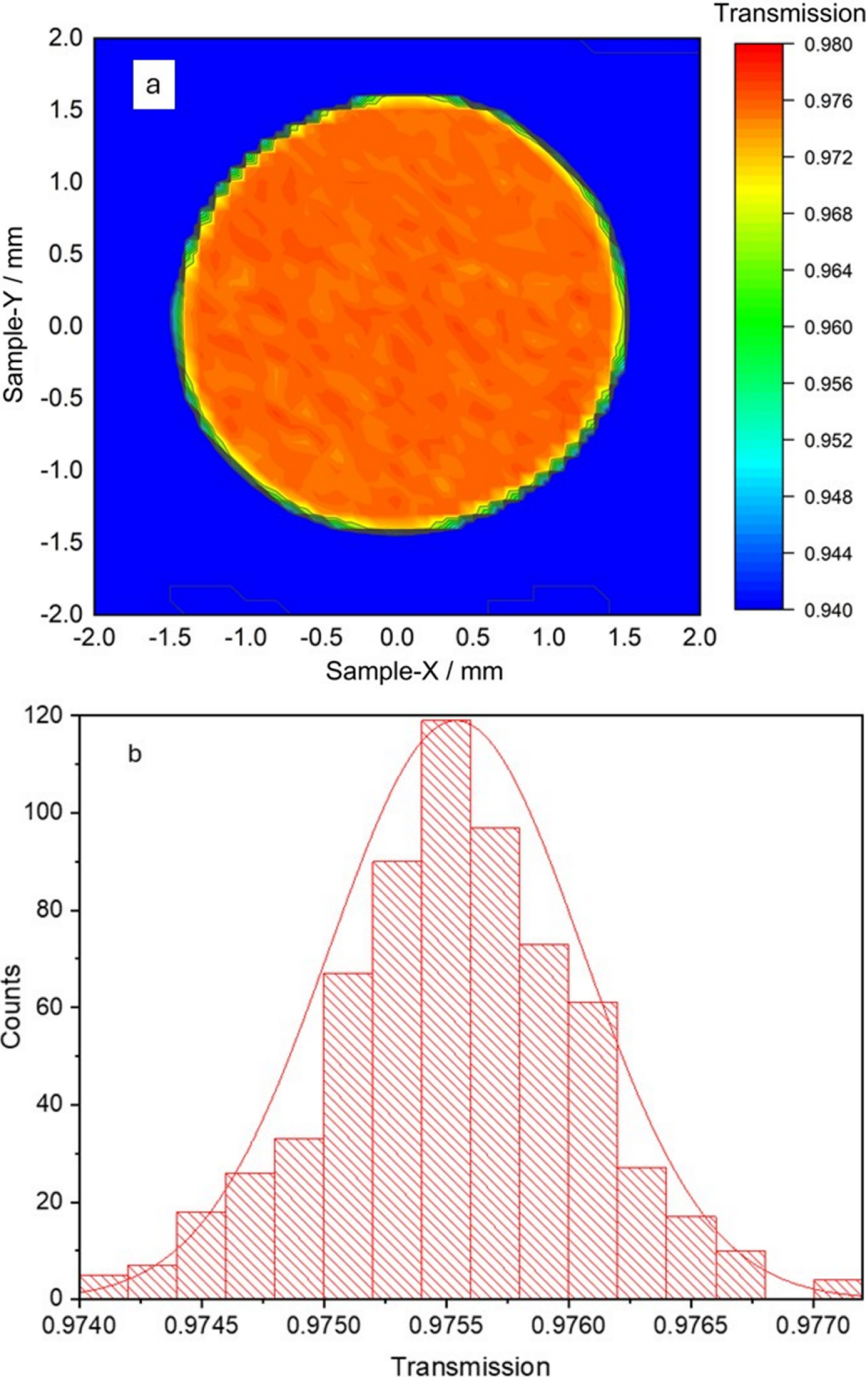
(*a*) Transmission map of the membrane. (*b*) Gaussian fit of the transmission distribution.

Fig. 4 ▸(*a*) shows a transmission map of the membrane area obtained performing a raster scan measurement with 8 keV photons. The results show a high degree of uniformity of the membrane surface. From a Gaussian fit of the transmission distribution [Fig. 4 ▸(*b*)], a mean transmission value of 97.5% is found, with a standard deviation of 0.05%. This result proves the low thickness variation of the membrane carved through the electrochemical etching procedure.

**Figure 5:**
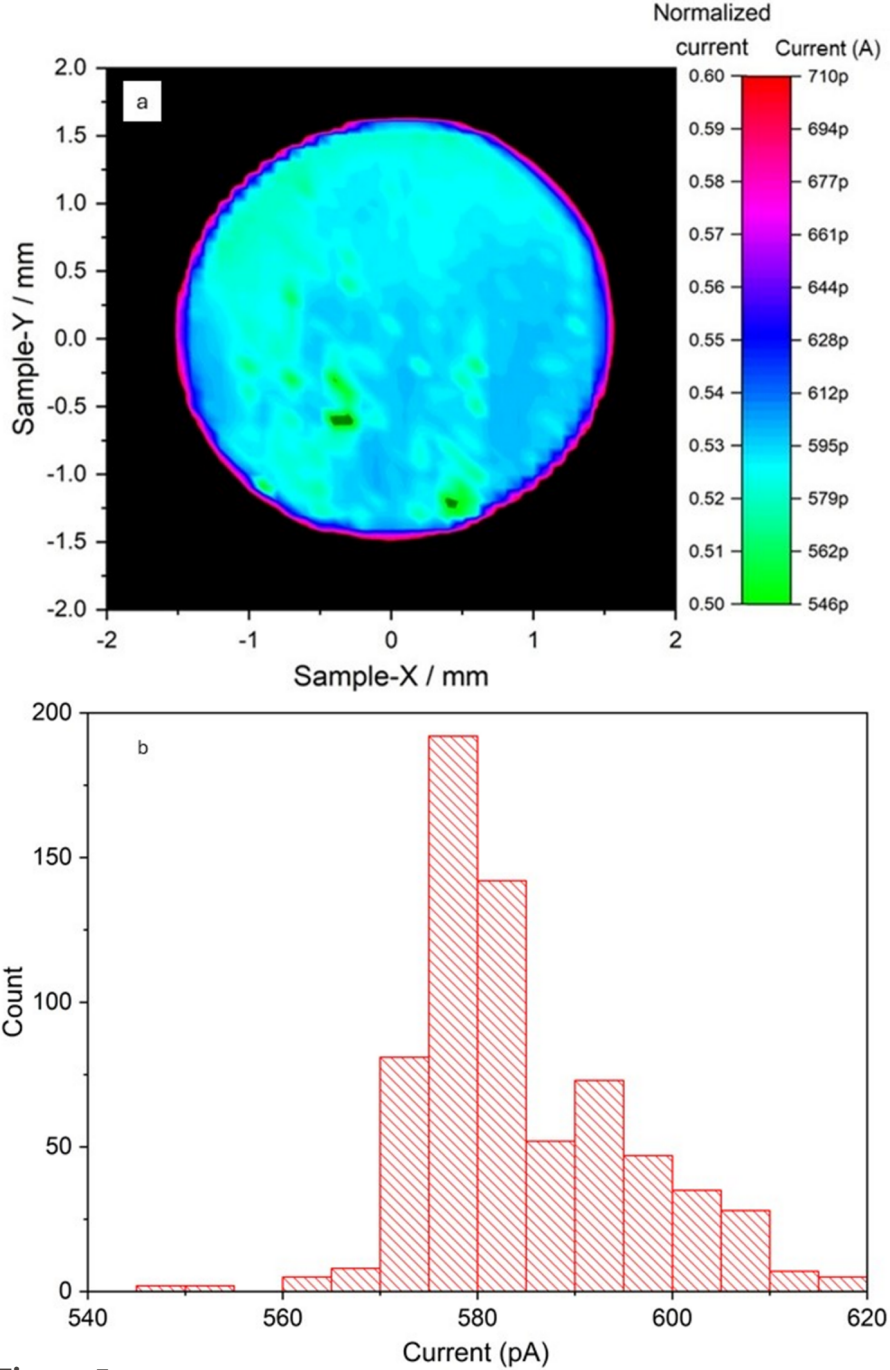
(*a*) 2D current map of the free-standing membrane. In the ‘normalized current’ scale, the signal is normalized to the current value measured in the region where the substrate is present. (*b*) Histogram of the current signal collected from the membrane region.

Fig. 5 ▸ reports the current signal measured by irradiating the device with 8 keV photons. The small dark-green spots represent areas in which the signal is lower than 500 pA, where there is probably a higher concentration of defects. The average current signal produced in the membrane is 0.58 nA at a photon flux of 1.89 × 10^8^ s^−1^. This value is in fairly good agreement with the expected current calculation from equation (2) (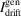 = 0.67 nA), considering an experimental CCE of about 86% (reported later). A signal uniformity on the membrane surface of about 2% is obtained as the standard deviation of the mean signal value. This low value indicates a high level of homogeneity in the detector response, which is crucial in beam intensity monitors.

The average current measured in the not-carved region is 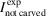 = 1.106 nA (*i.e.* about twice that of the one measured in the membrane). This value can be reproduced with the theoretical calculations reported in Section 2.2.

**Figure 6:**
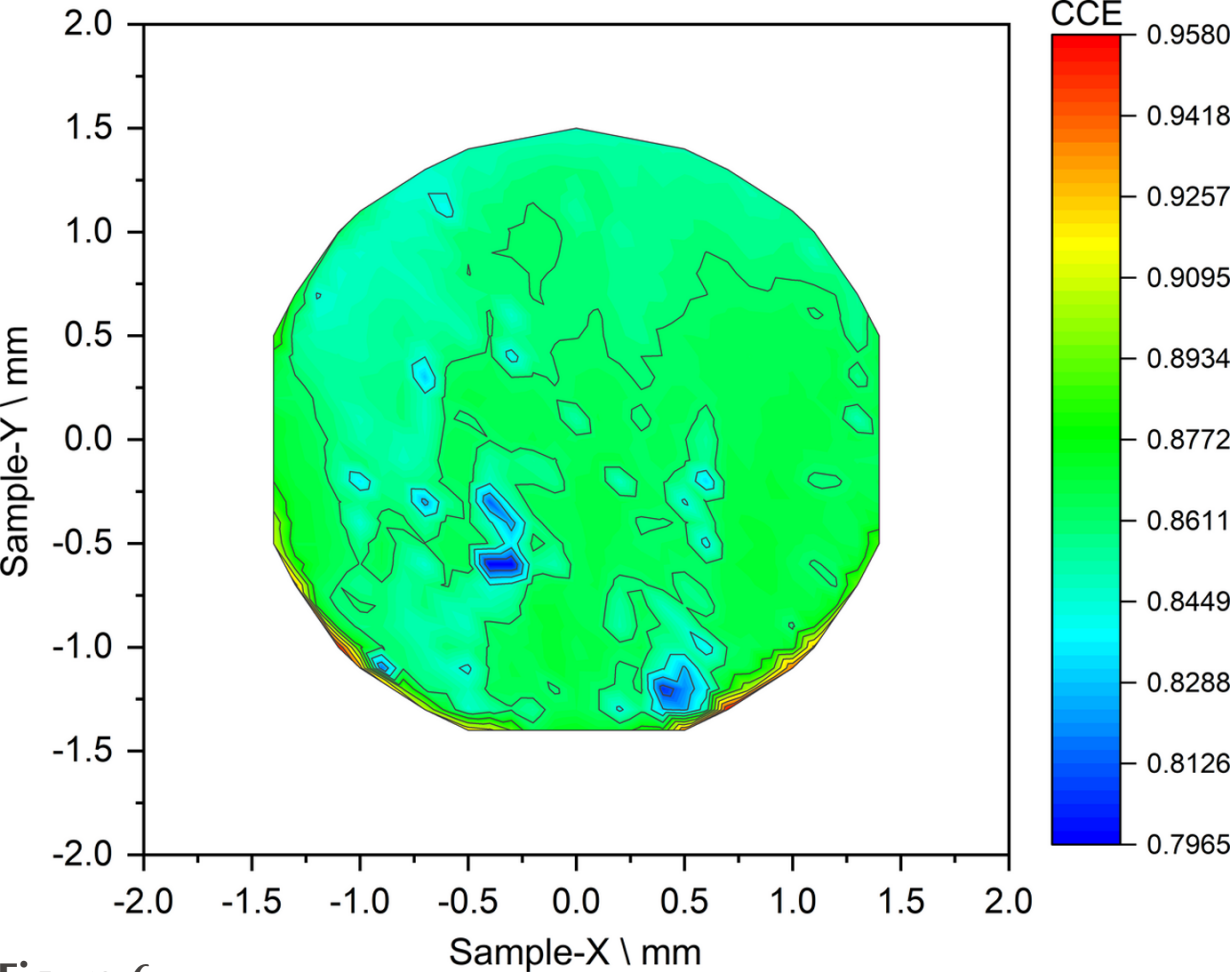
2D maps of the CCE of the SenSiC ‘free-standing membrane’ obtained with a raster scan using 8 keV X-rays.

The CCE of the device measured at 8 keV is shown in Fig. 6 ▸. It has been calculated as
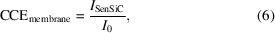
where *I*_SenSiC_ is the measured current and *I*_0_ = *en*_e−h_ is the theoretically produced current by the epitaxial n^−^ layer, as estimated by equation (2). The average CCE found on the membrane is 86%.

**Figure 7:**
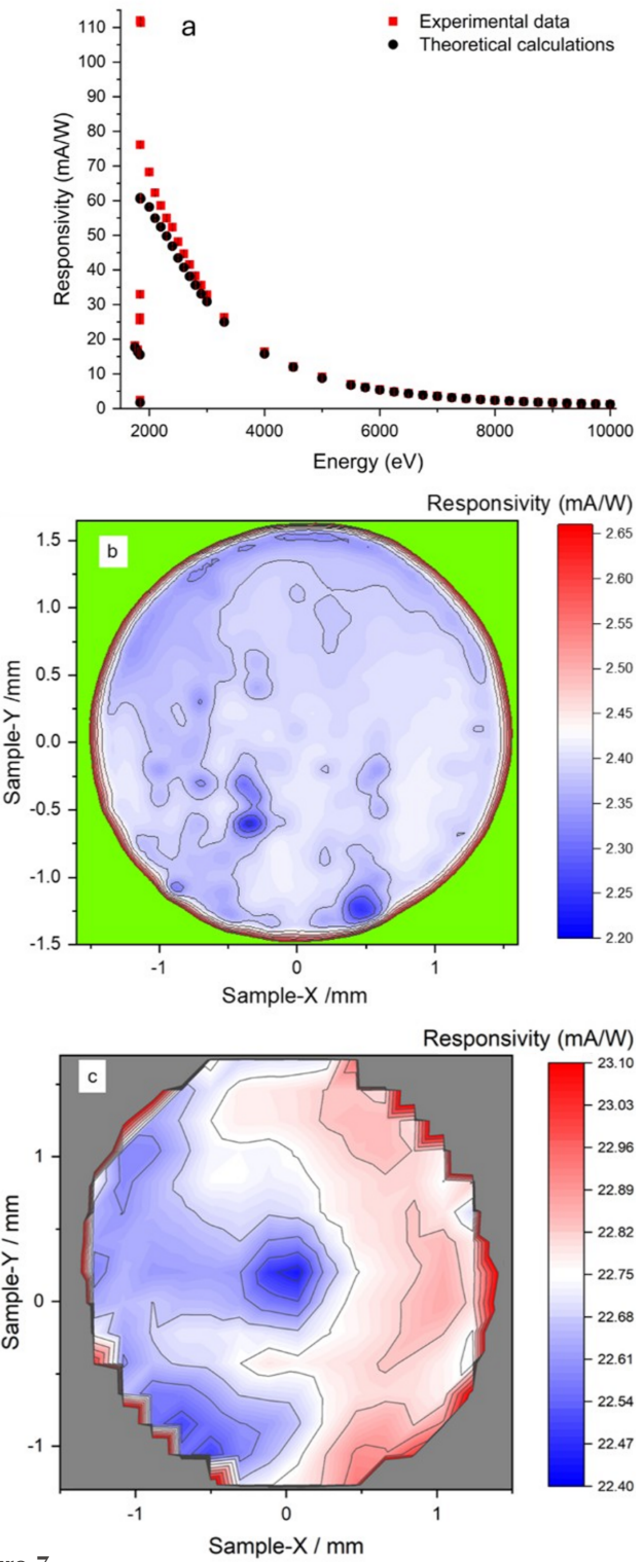
(*a*) Responsivity of the SenSiC diode irradiated in the central region of the membrane with X-rays from 1.75 keV to 10 keV. Experimental data are compared with the calculations (see text for details). Panels (*b*) and (*c*) show 2D maps of the responsivity of the diode irradiated with 8 keV and 3.5 keV X-rays, respectively.

Finally, the responsivity of the device (*i.e.* measured current over incident power) is shown in Fig. 7 ▸. The responsivity at various energies is obtained using the known responsivity of the calibrated Si thick diode (*R*_thick_), 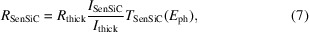
where *I*_thick_ is the measured current in the thick diode and *T*_SenSiC_ is the transmission of the thin membrane measured as a function of the photon energy. The responsivity as a function of the photon energy shows, as expected, a peak near the Si absorption *K*-edge [Fig. 7 ▸(*a*)]. Fig. 7 ▸(*a*) also shows the theoretical responsivity calculated using equation (2). The calculations demonstrate good agreement with the experimental results across the entire energy range, except in the *K*-edge region. Here, the experimental responsivity is greater than the theoretical one. This result reflects the fact that, as shown in Fig. 3 ▸ and previously discussed, the theoretical transmission calculated using absorption cross-sections from Henke *et al.* (1993 ▸) is higher than the measured one. Therefore, for the same incident power, the number of theoretically absorbed photons, and thus the signal, is lower than in the experimental case. Figs. 7 ▸(*b*) and 7 ▸(*c*) represent the responsivity maps of the membrane when it is irradiated with 8 keV and 3.5 keV, respectively. Values ranging from 22.40 mA W^−1^ to 22.90 mA W^−1^ and from 2.20 mA W^−1^ to 2.45 mA W^−1^ for 3.5 keV and 8 keV are found, depending on the region of the membrane hit by the beam. To calculate the responsivity of the not-carved area of the detector, it is not possible to use equation (7) directly because the beam is completely absorbed in this device region (*i.e.I*_thick_ = 0). However, by indirectly calculating the incident photon flux, it is possible to determine the responsivity of the unetched region as 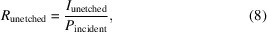
where *I*_unetched_ is the current measured in the unetched region and *P*_incident_ is the power of the photon flux incident on the device. This latter value is calculated using the information extracted from the membrane measurements. In particular, *P*_incident_ = 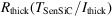, where *T*_SenSiC_ is the membrane transmission and *I*_thick_ is the current measured in the thick Si with the beam irradiating the carved region. The so-extracted average values of responsivity for the not-carved region are 38.33 mA W^−1^ and 4.47 mA W^−1^ for 3.5 keV and 8 keV X-rays, respectively.

### 3.2. State-of-the-art comparison

This section reports a comparative analysis of the SenSic free-standing membrane performance against various state-of-the-art Si- and diamond-based beam monitors.

The mean transmission values measured in the device studied in this work are 82% and 98.6% for photons of 4 keV and 10 keV, respectively. These high values indicate the device’s minimal interference with the beam, ensured by its high thinness. Despite this latter property, it is important to note that the measured membrane responsivity values (16.3 mA W^−1^ and 1.2 mA W^−1^ at 4 keV and 10 keV, respectively) are far from negligible and are in line with the state-of-the-art devices used for similar purposes (Table 1 ▸). Additionally, the CCE value obtained at 0 V, about 86%, is an excellent result considering that diamond devices reported in Table 1 ▸ have CCE values mostly greater than 90% (some even 100%), albeit achieved with bias applied. From these comparisons, it is clear that SiC free-standing membranes meet the requirement for advanced X-ray beam monitoring in synchrotron radiation facilities. All of this comes with the advantage, as mentioned earlier, of simpler scalability and lower production costs compared with diamond.

## 4. Conclusions

This study offers a comprehensive characterization of the SiC membrane devices produced by SenSiC GmbH. In particular, current signal, transmittance and responsivity of the device have been measured using X-rays at different energies in the PTB synchrotron laboratory at BESSY II. Moreover, an analytical model has been applied in order to reproduce the current signal from the not-carved part of the device. The high transmission (up to 98%), combined with a CCE of 86% at 0 bias, a responsivity comparable to the state-of-the-art sensors, designed for the same application, plus an excellent 2% signal uniformity, all demonstrate the optimal features of these innovative SiC membranes as beam intensity monitors. Considering the remarkable properties of SiC as a material, such as radiation hardness and high thermal conductivity, in addition to the exceptional thinness of the tested device, it is evident that these free-standing membranes offer an intriguing opportunity for use as beam monitors for tender and hard X-rays in synchrotron facilities.

## Figures and Tables

**Table 1:** Comparison of the SenSiC device with various temporarily available beam monitors in terms of thickness (*t*) transmittance (*T*) and responsivity (*R*) All data referring to commercial Si devices and the ion chamber are taken from Krumrey *et al.* (2007 ▸).

Manufacturer	*t* (µm)	*T*_4keV_ (%)	*T*_10keV_ (%)	*R*_4keV_ (mA W^−1^)	*R*_10keV_ (mA W^−1^)
4H-SiC(SenSiC), present work	1.8	82.0	98.6	16.3	1.2
Si (Sintef)	6.5	51.3	95.1	113.0	10.9
Si (Sintef)	11.8	29.0	91.1	159.2	19.4
Si (Sintef)	13.0	25.6	90.2	186.1	23.9
Si (Micron)	20.0	12.9	85.8	221.4	35.4
Si (IRD)	4.7	61.3	96.5	78.1	8.6
Si (Hamamatsu)	5.5	56.4	95.8	110.0	10.1
Si (Hamamatsu)	10.8	33.2	92.2	174.3	10.0
Ion chamber	50000†	53.0	93.0	12.3	0.8
scCVD (Desjardins *et al.*, 2013 ▸)	50.0	–	97.6	–	2.9
scCVD (Desjardins *et al.*, 2014 ▸)	3.0	96.5	–	3.1	–
scCVD (Keister *et al.*, 2018 ▸)	50.0	33.0	91.0	24.6	2.1
scCVD (Houghton *et al.*, 2023 ▸)	20.0	85.7	99.0	–	>0.62‡
4H-SiC (Houghton *et al.*, 2023 ▸)	2.3	82.0	98.3	–	>1.12‡

† The 50 mm ion chamber presents two 50 µm Be windows.

‡ The responsivity values are obtained using 12.4 keV X-rays.
